# In vitro effects of platelet-rich gel supernatants on histology and chondrocyte apoptosis scores, hyaluronan release and gene expression of equine cartilage explants challenged with lipopolysaccharide

**DOI:** 10.1186/s12917-016-0759-8

**Published:** 2016-07-01

**Authors:** Jorge U. Carmona, Diana L. Ríos, Catalina López, María E. Álvarez, Jorge E. Pérez, Mabel E. Bohórquez

**Affiliations:** Grupo de Investigación Terapia Regenerativa, Departamento de Salud Animal, Universidad de Caldas, Manizales, Colombia; Grupo de investigación Biosalud, Departamento de Ciencias Básicas para la Salud, Universidad de Caldas, Manizales, Colombia; Grupo de Investigación en Citogenética, Filogenia y Evolución de Poblaciones, Universidad del Tolima, Ibagué, Colombia

**Keywords:** Platelet-rich plasma, Cartilage explants, Chondrocyte apoptosis, Catabolic/anabolic gene expression, Growth factors

## Abstract

**Background:**

Platelet-rich plasma (PRP) preparations are a common treatment in equine osteoarthritis (OA). However, there are controversies regarding the ideal concentration of platelets and leukocytes in these biological substances necessary to induce an adequate anti-inflammatory and anabolic response in articular cartilage. The aims were to study the influence of leukocyte- and platelet-rich gel (L-PRG) and pure platelet-rich gel (P-PRG) supernatants on the histological changes of cartilage, the degree of chondrocyte apoptosis, the production of hyaluronan (HA) and the gene expression of nuclear factor kappa beta (NFkβ), matrix metalloproteinase 13 (MMP-13), a disintegrin and metalloproteinase with thrombospondin motifs 4 (ADAMTS-4), collagen type I alpha 1 (COL1A1), collagen type II alpha 1 (COL2A1) and cartilage oligomeric matrix protein (COMP) in normal cartilage explants (CEs) challenged with lipopolysaccharide (LPS).

**Results:**

Overall, 25 % L-PRG supernatant (followed in order of importance by, 50 % P-PRG, 25 % P-PRG and 50 % L-PRG) represented the substance with the most important anti-inflammatory and anabolic effect. 25 % P-PRG supernatant presented important anabolic effects, but it induced a more severe chondrocyte apoptosis than the other evaluated substances.

**Conclusions:**

25 % L-PRG supernatant presented the best therapeutic profile. Our results demonstrate that the biological variability of PRP preparations makes their application rather challenging. Additional in vivo research is necessary to know the effect of PRP preparations at different concentrations.

## Background

Osteoarthritis (OA) is a chronic inflammatory disease that affects middle aged and older people worldwide [1]. This disease also affects horses and other animal species, which causes economic losses by diminishing the athletic potential of the affected animals and through the chronic use of anti-inflammatory drugs and nutraceuticals [2–4]. Thus, the horse is an important animal model to study both the pathophysiological mechanisms and the experimental treatments of OA [5, 6].

There are several extrinsic factors associated with OA development such as joint repetitive trauma, obesity, joint infection and osteochondrosis, amongst others [4]. During the OA developmental process, there are structural (histological) changes indicating the severity or degree of affectation of the articular cartilage [7]. This includes increased chondrocyte necrosis, cluster (complex chondrone) formation, fibrillation/fissuring, focal cell loss and diminished uptake of safranin-O fast green (SOFG) stain [8]. Furthermore, another important histological change associated with OA is chondrocyte apoptosis, which leads to loss of resident cells in the articular cartilage and results in severe alterations such as impaired extracellular matrix (ECM) remodelling [9]. This phenomenon has been described in both human and animal models of OA [10, 11]. Chondrocyte apoptosis could be detected by caspase-3 expression or terminal deoxynucleotidyl transferase dUTP nick end labelling (TUNEL) staining [12].

Together with the OA histological changes are several joint molecular alterations characterized by nuclear factor kappa beta (NFkβ) upregulation [13] that subsequently upregulates several catabolic and proinflammatory cytokines for cartilage such as interleukin-1 (IL-1) and tumour necrosis factor alpha (TNF-α), matrix metalloproteinases (MMPs - e.g. MMP-13), aggrecanases (i.e. a disintegrin and metalloproteinase with thrombospondin motifs 4 - ADAMTS-4), inducible synthases of nitric oxide and cyclooxygenase type 2 [14]. Consequently, a proinflammatory molecular milieu produces histological alterations in cartilage and induces molecular changes with clinical accompanying indicators such as pain and synovial effusion [4].

In the initial OA stages, joint tissues (e.g. cartilage) act by upregulating anabolic gene expression to induce cartilage ECM anabolism and to increase the synthesis of collagen type II alpha 1 (COL2A1), cartilage oligomeric matrix protein (COMP) and hyaluronan (HA) amongst other anabolic ECM components. However, as the OA progresses, the local expression and production of collagen type I alpha 1 (COL1A1) appears as a poor anabolic response by the osteoarthritic cartilage [15, 16].

Platelet-rich plasma (PRP) has been used as a therapy for OA in humans [17–19] and animals [20–23]. The rationale for the therapeutic use of PRP stems from the fact that platelets (PLTs) (but also white blood cells –WBC-) contained in PRP represent an important autologous source of some growth factors (GFs), which include transforming growth factor beta 1 (TGF-β_1_), platelet derived growth factor isoform BB (PDGF-BB) [24, 25], and other anti-inflammatory cytokines such as interleukin 4 (IL-4) and the receptor antagonist of IL-1 (IL-1ra) [26, 27], which are implicated in the anabolic characteristics of OA [4, 28]. Furthermore, in vitro studies have revealed that PDGF-BB downregulates NFkβ [29, 30] and increases the proliferation of chondrocytes [29, 31], whereas TGF-β_1_ increases the cartilage extracellular matrix (ECM) synthesis and promotes the differentiation of stem cells in chondrocytes [32].

Currently, there is controversy about which PRP preparation is ideal for the intra-articular treatment of patients with OA [33, 34]. Some in vitro [35–37] and clinical studies have suggested that leukoreduced PRP (also known as pure PRP [P-PRP]) preparations have better anabolic and anti-inflammatory effects for OA treatment than leukoconcentrate PRP (L-PRP) preparations, whereas other studies have described similar positive effects for both preparations [32, 38, 39].

Recently, we demonstrated in an in vitro system of cartilage inflammation that 50 % L-PRP releasate produced a more sustained temporal concentration of growth factors and anti-inflammatory cytokines than both 25 % L-PRP and P-PRP releasates and 50 % P-PRP releasate in relation to the concentration in culture media [40]. However, there is no information regarding the effects of these substances on the histology or ECM cartilage gene expression.

The study presented here shows complementary and novel information for the anabolic, anti-inflammatory effect of two supernatant concentrations (25 and 50 %) from both P-PRP and L-PRP preparations on equine cartilage explants (CEs) challenged with lipopolysaccharide (LPS). This research evaluates and compares the influence of both platelet-rich gel (PRG) supernatants on the histological changes of cartilage, the degree of chondrocyte apoptosis, the production of HA and the gene expression of NFkB, MMP-13, ADAMTS-4, COL1A1, COL2A1 and COMP in an in vitro system of cartilage inflammation and degeneration.

The hypothesis of this study is that L-PRG supernatants at a lower concentration (25 %) have better anti-inflammatory and anabolic effects than both PRG supernatants at the higher 50 % concentration and P-PRG supernatant at 25 % concentration in CEs challenged with LPS.

## Methods

This study was approved by the committee on animal experimentation of the Universidad de Caldas, Manizales, Colombia.

### Samples

Cartilage samples from the metacarpophalangeal joints from six horses, aged 4 to 9 years, were included in this study. The samples were taken from horses that were free from musculoskeletal disease and euthanised by a pentobarbital intravenous overdose for other medical reasons. All metacarpophalangeal joints were radiographed and macroscopically evaluated to exclude horses with OA-associated joint changes.

### L-PRP and P-PRP preparation

**Fig. 1:**
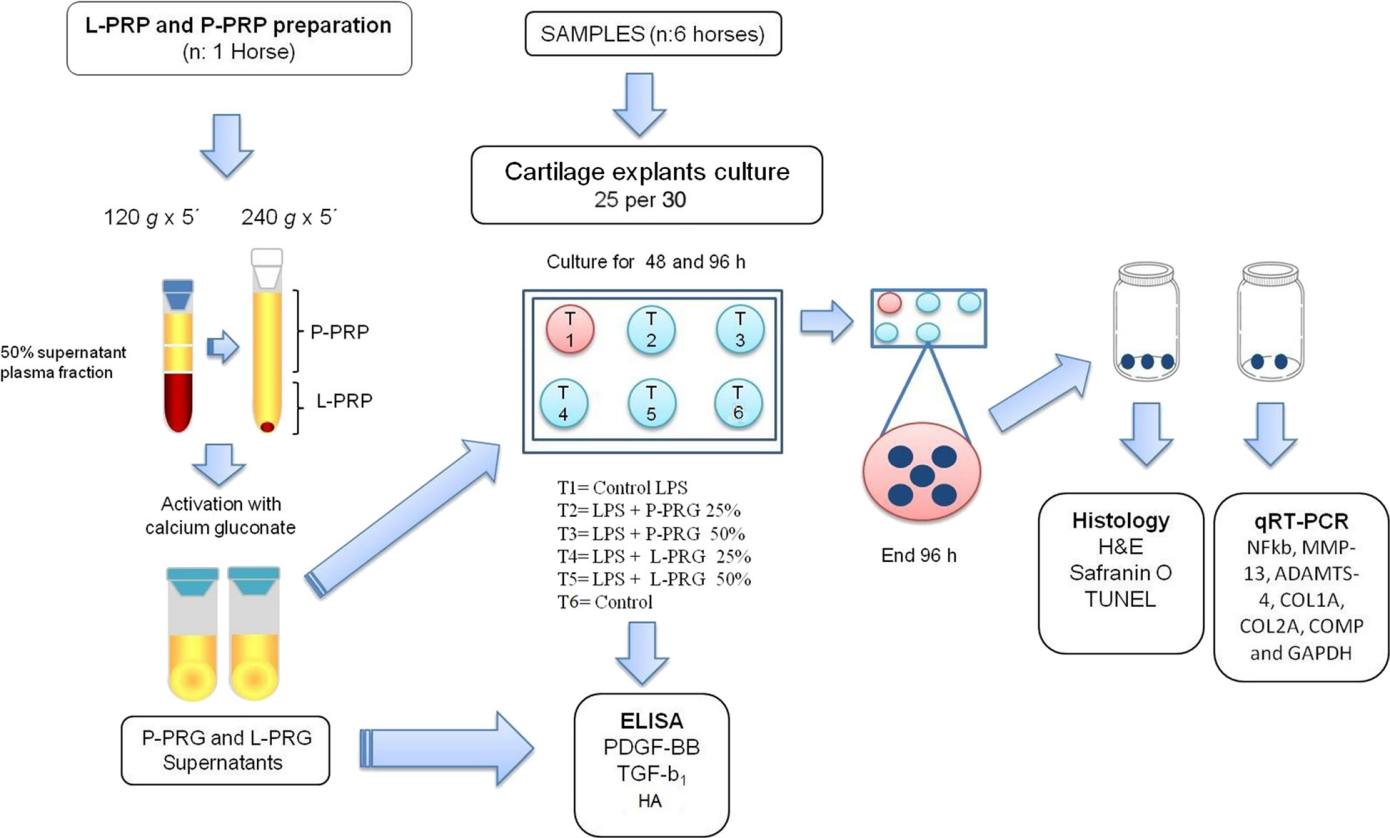
Schematic workflow of the experiments of the study

Venous blood from one clinically healthy mare was used in order to avoid the great variability in the GF and cytokine concentrations in the PRG supernatants used in the experiments. Platelet concentrates were obtained through a manual double centrifugation tube method [41] that was previously validated and used clinically in horses with OA [21]. Blood was drawn from jugular venipuncture and immediately deposited in 4.5 mL tubes with sodium citrate solution (BD Vacutainer®, Becton Drive, Franklin Lakes, NJ, USA). After centrifugation at 120 × *g* for five minutes, the first 50 % of the top supernatant plasma fraction, adjacent to the buffy coat, was collected. This fraction was centrifuged at 240 × *g* for five minutes, and then the bottom quarter of the fraction was collected [41]. This fraction was considered as L-PRP. The upper plasma fraction was considered as P-PRP (Fig. 1). Whole blood and both PRP preparations were analysed for PLT and WBC counts using an impedance-based haematology device (Celltac-α MEK 6450, Nihon Kodhen, Japan).

Both PRP preparations were activated with calcium gluconate (Ropsohn Therapeutics Ltda®, Bogotá, Colombia) (ratio 1:10) and incubated at 37 °C for 1 h until clot retraction occurred. Fresh L-PRG and P-PRG supernatants were used to add to the culture media at two concentrations (25 % and 50 %). Aliquots of both PRG supernatants obtained were frozen at -86 °C for later quantification of PDGF-BB, TGF-β_1_ and HA.

### Cartilage explants culture and LPS challenge

Cartilage samples were obtained aseptically, and circular 4 mm diameter explants were obtained using a disposable biopsy punch (KAI Medical, Solingen, Germany). CEs were dissected from the articular surface without calcified cartilage layers and washed in phosphate-buffered saline.

CEs were stabilised in Dulbecco’s Modified Eagle Medium (DMEM) (high glucose, 4500 mg/L) with L-glutamine and sodium bicarbonate and free of sodium pyruvate (DMEM, Lonza Group Ltd, Basel, Switzerland) and supplemented with streptomycin (100 μg/mL) and penicillin (100 μg/mL) without the addition of serum. Cultures were incubated in a 5 % CO_2_ and water saturated atmosphere for 24 h and then replaced with fresh culture media. At this time point (24 h), the CEs were challenged with 100 ng/mL of LPS (Sigma-Aldrich, St Louis, MO, USA) to induce inflammatory/catabolic damage of the cartilage [42, 43].

### Study design

A total of 30 CEs were obtained from each horse. The study design included the evaluation of six experimental groups using five CEs per group from each horse as follows: one CE healthy control group without LPS and without addition of any PRG supernatant, one CE control group challenged with LPS and without addition of any PRG supernatant, and four CE groups cultured with L-PRG and P-PRG supernatants at two different concentrations (25 % and 50 %). After 1 h of incubation, L-PRG and P-PRG supernatants were added in order to obtain concentrations at 25 and 50 %. All CE groups were cultured at 96 h, after which the culture media were frozen in aliquots at -86 °C for later determination of HA concentration by ELISA. Furthermore, the CEs were deposited in buffered formaldehyde for histological analysis or in an RNA conserving solution (RNAlater, Life Technologies, Carlsbad, CA, USA) for quantitative gene expression of NFkB, MMP-13, ADAMTS-4, COL1A1, COL2A1 and COMP. The schematic diagram (Fig. 1) summarises the study design and methodology.

### ELISA analysis

L-PRG and P-PRG supernatants and culture media at 96 h were used to determine the concentration of PDGF-BB, TGF-β_1_ and HA via ELISA in duplicate. All proteins were assayed using commercial ELISA development kits from R&D Systems (Minneapolis, MN, USA). PDGF-BB (Human PDGF-BB DuoSet, DY220) and TGF-β_1_ (Human TGF-β1 DuoSet, DY240E) were determined using human antibodies because there is a high sequence homology between these proteins in humans and horses [44, 45]. Furthermore, these kits have been used for the same purposes in other equine PRP studies [24, 25]. HA (Hyaluronan, DuoSet, DY3614) was determined using a multispecies detection ELISA kit. Standards provided for each ELISA kit were used to prepare each standard curve following the manufacturers’ instructions. Absorbance readings were performed at 450 nm.

### H&E histology evaluation

CEs were dehydrated in serial alcohol concentrations, fixed in wax blocks, cut into 3 μm slices and stained with either haematoxylin and eosin (H&E) or safranin O stains. The outcome parameters evaluated were: (1) chondrocyte necrosis, (2) cluster (complex chondrone) formation, (3) fibrillation/fissuring, (4) focal cell loss and (5) safranin-O stain uptake. All preparations were analysed using a light microscope in a 40× magnification following the OARSI histopathology initiative for histological assessments of osteoarthritis in the horse [8] (Table 1).

**Table 1:** Microscopic grading system for articular cartilage histology [8]

Articular cartilage		
Outcome parameter	Score	Description
Chondrocyte necrosis*	0	Normal section without necrosis
	1	No more than one necrotic cell located near the articular surface per 20x objective
	2	1e2 necrotic cells located near the articular surface per 20x objective
	3	2e3 necrotic cells located near the articular surface per 20x objective
	4	3e4 necrotic cells located near the articular surface per 20x objective
Cluster (complex chondrone) formation	0	No cluster formation throughout section
	1	Two chondrocytes (doublets) within same lacunae along superficial aspect of the articular cartilage section
	2	2e3 chondrocytes (doublets & triplets) within same lacunae along superficial aspect of the articular cartilage section
	3	3e4 chondrocytes within same lacunae along superficial aspect of the articular cartilage section
	4	Greater than four chondrocytes within same lacunae along superficial aspect of the articular cartilage section
Fibrillation/fissuring	0	No fibrillation/fissuring of the articular cartilage surface
	1	Fibrillation/fissuring of the articular cartilage restricted to surface and superficial zone
	2	Fissuring that extends into the middle zone
	3	Fissuring that extends to the level of the deep zone
	4	Fissuring that extends into the deep zone
Focal cell loss*	0	Normal cell population throughout the section
	1	A 10e20% area of acellularity per 20x field
	2	A 20e30% area of acellularity per 20x field
	3	A 40e50% area of acellularity per 20x field
	4	A greater than 50 % area of acellularity per 20x field
Safranin-O stain uptake	0	Normal staining
	1	Less than 25 % loss of staining characteristics
	2	25e50% loss of staining characteristics
	3	50e75% loss of staining characteristics
	4	Greater than 75 % loss of staining characteristics

*Chondrocyte necrosis is used to grade presence of lacunae with necrotic nuclei still present compared to focal cell loss which is most likely an extension of the pathologic change but lacunae or nuclei are no longer present

These variables were quantified using a 0–4 scale, with 0 being normal and 4 being abnormal (Table 1). Thus, a totally normal CE would score 0 and a maximally abnormal CE would score 15 [8]. Three sections randomly selected from each sample were evaluated blindly and the average score was used for comparison.

### TUNEL assay for chondrocyte apoptosis

TUNEL staining was performed to detect DNA strand breakage using a commercial kit for cell and tissues (TACS TdT-Blue Label In situ Apoptosis Detection Kit, Trevigen, Inc., Gaitherburg, MD, USA). Briefly, the CEs were washed and dehydrated in serial alcohol concentrations, fixed in wax blocks and cut into 3 μm slices. The tissue sections were deparaffinised with xylene and ethanol and washed with PBS. The samples were covered with 50 μl of proteinase K solution and incubated for 15 min at 37 °C in a humidity chamber. The samples were washed with PBS and covered with 50 μl of labelling reaction mix. After this, the samples were immersed in a stop buffer solution for 5 min at room temperature to stop labelling reaction and once more washed twice with PBS for 2 min at room temperature. The samples were covered with 50 μl of antibody solution and incubated for 30 min at 37 °C then washed with PBS and covered with 50 μl of TACS Blue Label™ (Trevigen, Inc., Gaitherburg, MD, USA). These samples were washed with deionised water and counterstained with nuclear fast red. The controls were treated similarly except that terminal deoxynucleotidyl transferase was substituted by distilled water [46].

After the reaction, stained chondrocytes (grey-black) were analysed under a light microscope. Chondrocyte apoptosis was scored by a semiquantitative approach using a 0-4 scale, where 1 = 6–25 % of chondrocyte apoptosis; 2 = 26–50 % of chondrocyte apoptosis; 3 = 51–75 % of chondrocyte apoptosis and 4 = 76–100 % of chondrocyte apoptosis [47].

### Molecular evaluation

CE samples were pulverised in liquid nitrogen and mixed with TRIzol reagent (Life Technologies, Carlsbad, CA, USA) for 5 min. The samples were centrifuged for 10 min at 10,000 × g, and the supernatant was mixed with 20 % chloroform (volume/volume) and then centrifuged for 15 min at 12,000 × g. The aqueous phase of the samples was removed and transferred to special columns (PureLink RNA Mini Kit, Life Technologies, Carlsbad, CA) for RNA extraction according to the manufacturer’s instructions. RNA concentrations were measured using a spectrophotometer (Nanodrop 2000, Thermo Scientific, Wilmington, DE, USA). Samples were diluted to a concentration of 5 ng/μL of RNA.

The samples were assayed for quantitative gene expression levels in a qRT-PCR device (StepOnePlus Real-Time PCR System, Life Technologies, Carlsbad, CA, USA) using a SuperScript III platinum SYBR Green One-Step qRT-PCR kit (Life Technologies, Carlsbad, CA, USA). Primers for NFkB, MMP-13, ADAMTS-4, COL1A1, COL2A1, COMP and GAPDH (glyceraldehyde 3-phosphate dehydrogenase) were designed and validated in the Colombian Centre for Bioinformatics and Computational Biology (BIOS) (Table 2).

**Table 2:** Genes and sequence of primers evaluated in the study

Targeted genes	Primer sequences (5'- > 3')	Product size (bps)
GAPDH, glyceraldehyde-3-phosphate dehydrogenase 6	Forward TCCCTGCTTCTACTGGTGCT Reverse TGACAAAGTGGTCGTTGAGG	306
NFkβ, nuclear factor of kappa light polypeptide gene enhancer in B-cells-like 1	Forward CGATTTCGATATGGCTGTGA Reverse CACCTTCTTCAGCTCCTTGG	399
MMP 13, matrix metallopeptidase 13 (collagenase 3) 7	Forward GCATTCAAAAAGGCCTTCAA Reverse GGAAGCACAAAGTGGCTTTT	356
ADAMTS 4, metallopeptidase with thrombospondin type 1 motif 4	Forward TGTCAGCTTGGTGGTGACTC Reverse GTTGAAGACATGGCCCAGTT	322
COL1A1, collagen, type I, alpha 1 11	Forward AGCCAGCAGATCGAGAACAT Reverse CTGGCCACCATACTCGAACT	309
COL2A1, collagen, type II, alpha 1 6	Forward primer ACGTCCAGATGACCTTCCTG Reverse primer GTCCACACCAAATTCCTGCT	326
COMP, cartilage oligomeric matrix protein 21	Forward CCACGTGAATACGGTCACAG Reverse TAGGAACCAGCGGTAGGATG	301

The relative change in gene expression was determined via the comparative 2^-ΔΔ*C*T^ method [48]. GAPDH was used as the internal control (housekeeping gene), and cartilage samples from all horses that were not incubated with any treatment were used as reference samples.

### Statistical and data analysis

The statistical analysis was performed with SPSS 19.0 software (IBM, Chicago, IL, USA). The Shapiro–Wilk test was used to assess the fit of the data set to a normal distribution (goodness of fit). All the parameters evaluated, except CE histology and apoptosis scores, demonstrated a normal distribution (*p* > 0.05). However, these data were normalised by using a transformation log (Y).

Platelet and WBC counts in whole blood and both PRP preparations were evaluated through a one-way analysis of variance (ANOVA), followed by a Tukey test. PDGF-BB, TGF-β_1_ and HA concentrations from both PRG supernatants were compared using a t-non-paired test. A generalised lineal model (GLM), followed (when necessary) by a Tukey test, was used for comparing HA concentrations in culture media and gene expression at 96 h. The HA concentrations in fresh culture media with PRG supernatants at 1 h and 96 h were compared using a t-paired test. A *p* < 0.05 was accepted as statistically significant for all tests. Data are presented as mean ± mean standard error (m.s.e).

## Results

### Cell, growth factor and HA in L-PRP/L-PRG and P-PRP/P-PRG

PLT counts were significantly (*p* < 0.05) different between whole blood, L-PRP and P-PRP, with the lowest concentration for P-PRP (98.7 00 ± 4.600 PLT/μL), followed by whole blood (125.900 ± 3.400 PLT/μL) and L-PRP (312.800 ± 19.600 PLT/μL). WBC counts were also significantly different between the evaluated groups, with a higher concentration for L-PRP (35.100 ± 3.500 WBC/μL), followed by whole blood (8.300 ± 3.700 WBC/μL) and P-PRP (110 ± 40 WBC/μL). TGF-β_1_ concentration was similar between L-PRG (1676.3 ± 312.4 pg/mL) and P-PRG (1356.9 ± 20.8 pg/mL). PDGF-BB had a significantly (*p* < 0.05) higher concentration in L-PRG (3053. 8 ± 956.7 pg/mL) when compared to P-PRG (372.7 ± 79.6 pg/mL). HA concentration was significantly (*p* < 0.05) higher in L-PRG supernatant (4.3 ± 1.7 pg/mL) in comparison to P-PRG supernatant (1.1 ± 0.21 pg/mL).

### H&E histology evaluation

**Fig. 2:**
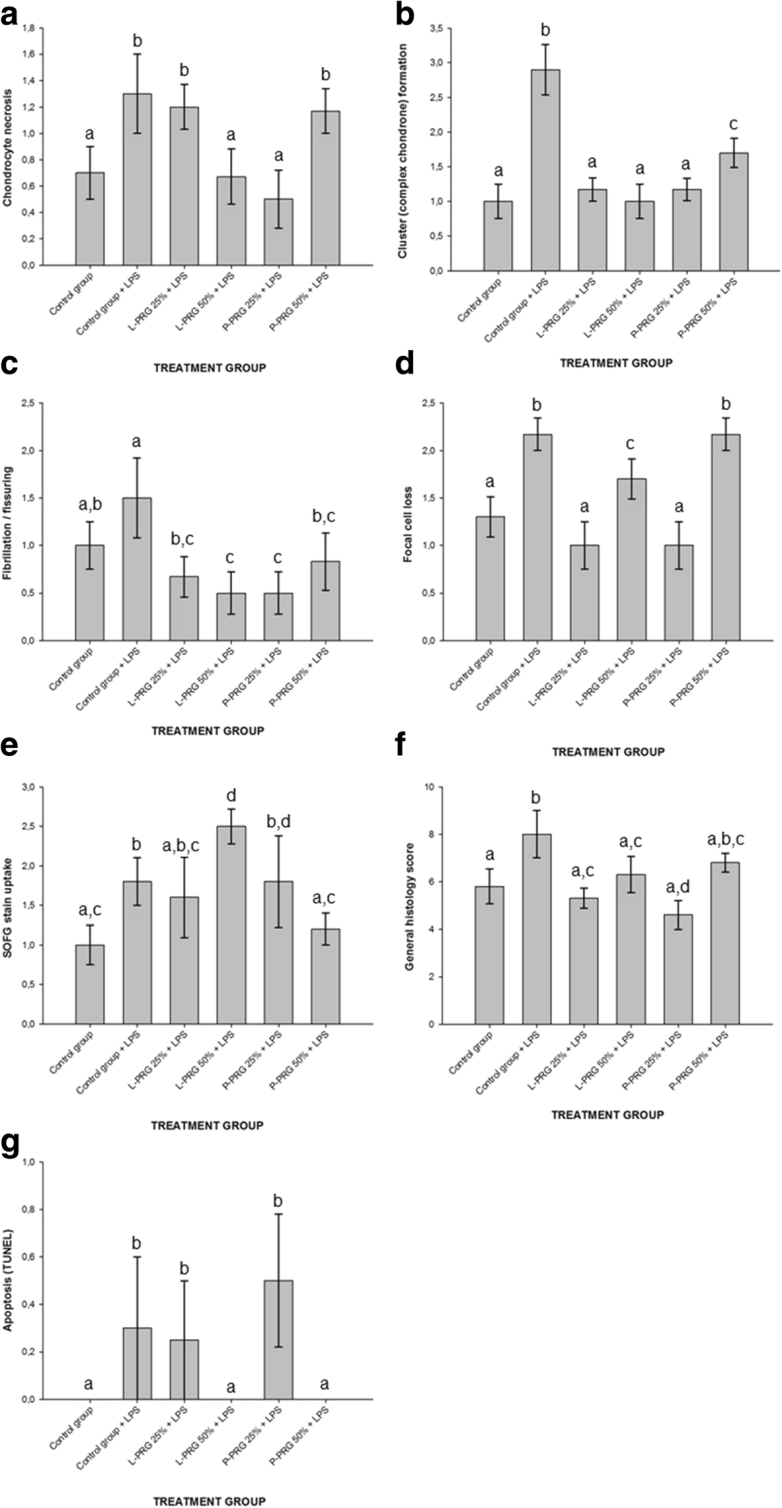
Means (±standard error of the mean [s.e.m]) of the cartilage histology and chondrocyte apoptosis scores. **a** Chondrocyte necrosis. **b** Cluster formation. **c** Fibrillation/fissuring. **d** Focal cell loss. **e** SOFG stain uptake. **f** Total histology score. **g** Chondrocyte apoptosis. ^a-b^Different lowercase letters denote significant (*p* ˂ 0.05) differences between the groups evaluated by the Tukey test

Chondrocyte necrosis scores were significantly (*p* < 0.05) worse (higher) for CEs from the control group with LPS and those treated with 25 % L-PRG and 50 % P-PRG supernatants when compared to CEs of the control group and those treated with 50 % LPRG and 25 % P-PRG supernatants (Fig. 2a). Cluster formation was significantly higher for CEs from the control group plus LPS when compared to the rest of the CE groups evaluated. This histology parameter was similar among CEs from the healthy control group and those treated with both L-PRG supernatants and 25 % P-PRG supernatant, whereas CEs treated with 50 % P-PRG supernatant presented a significantly worse score than the aforementioned CE groups (Fig. 2b).

Fibrillation/fissuring scores were significantly (*p* < 0.05) higher in both control groups, although there was a statistical trend (*p* = 0.10) showing CEs of the healthy control group with lower score in comparison to CEs of the control group treated with LPS. In general, CEs treated with all PRG supernatants presented significantly (*p* < 0.05) lower scores. However, CEs treated with 50 % L-PRG and 25 % P-PRG supernatants were significantly lower than CEs of healthy control group (Fig. 2c).

Focal cell loss scores were significantly (*p* < 0.05) worse for the CEs of control group treated with LPS and those treated with 50 % P-PRG supernatant. CEs from the healthy control group and those treated with both 25 % PRG supernatants presented significantly (*p* < 0.05) lower scores than the rest of the CE groups (Fig. 2d). SOFG stain uptake score were significantly (*p* < 0.05) worse for CEs treated with 50 % L-PRG supernatant, followed by CEs of the control group challenged with LPS and both 25 % PRG supernatants. The best score for this histology parameter was found in CEs of the healthy control group and those treated with 50 % P-PRG supernatant (Fig. 2e).

The general histology total score was significantly (*p* < 0.05) higher in CEs of the control group challenged with LPS when compared to CEs of the healthy control group and those treated with both L-PRG and 25 % P-PRG supernatant. In general, CEs treated with both 25 % PRG supernatants showed significantly (*p* < 0.05) better general histology score than the rest of the groups evaluated (Fig. 2f).

### TUNEL assay for chondrocyte apoptosis

No chondrocyte apoptosis was detected in CEs of the healthy control group and those treated with both 50 % PRG supernatants. However, this cellular phenomenon was detected (and it was significantly higher [*p* < 0.05]) in CEs of the control group challenged with LPS and those treated with both 25 % PRG supernatants; although, the score for this parameter was apparently lower for those CEs treated with 25 % L-PRG supernatant (Fig. 2g).

### Concentration of HA in culture media at 1 h and 96 h

**Fig. 3:**
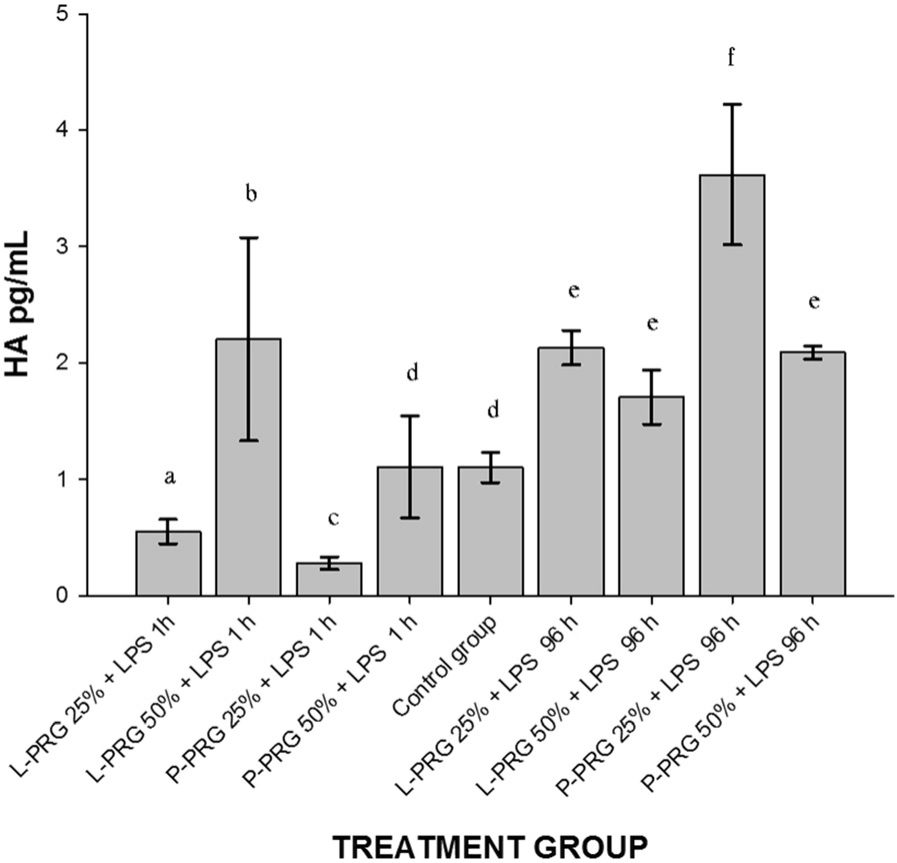
Means (± s.e.m) of HA concentration in culture media from cartilage explant (CE) groups at 1 h and 96 h. ^a-b^ Different lowercase letters denote significant (*p*  <  0.05) differences between the groups evaluated by the Tukey test over the same period of time. * Denotes significant differences between the same variable at different time periods by the t-paired test

At 1 h, HA concentrations varied significantly (*p* < 0.05) between all PRG supernatant groups evaluated. At 96 h, HA was released by all CE groups. However, the CE group cultured with 25 % P-PRG supernatant presented the highest significant (*p* < 0.05) HA release in comparison with the CE control group and the other CE PRG-treated groups. Notably, HA concentrations were different between CE PRG treated groups at 1 h and 96 h (Fig. 3).

### Gene expression

**Fig. 4:**
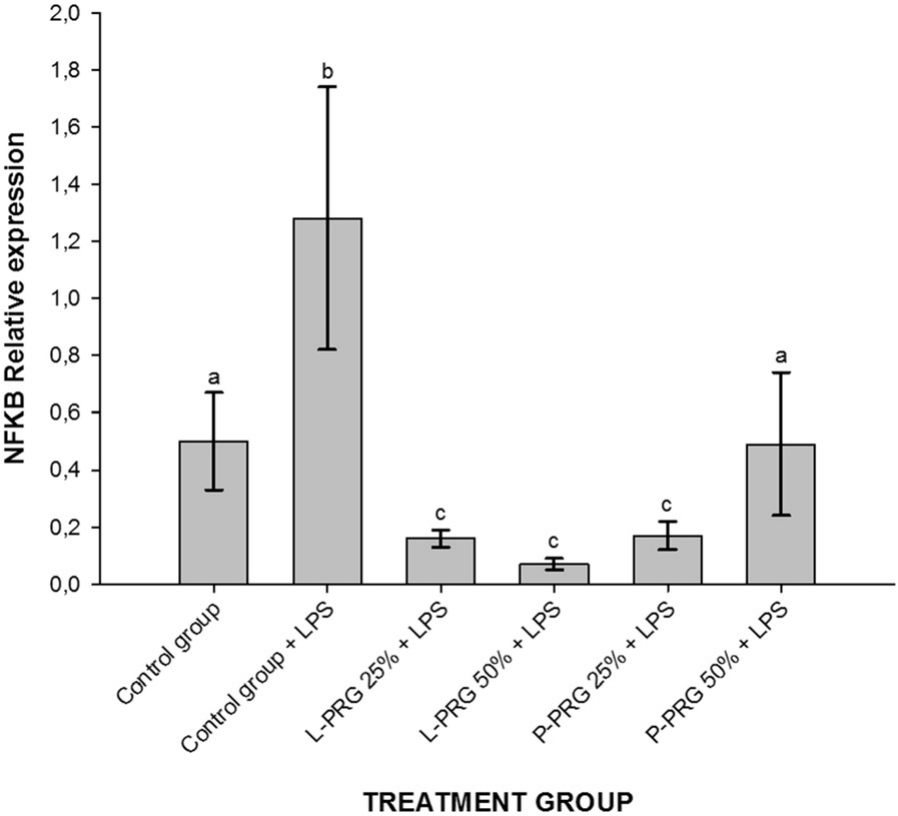
Means (± s.e.m) of NFkB gene expression in CE groups at 96 h. ^a-b^ Different lowercase letters denote significant (*p* < 0.05) differences between the groups evaluated by the Tukey test

NFkB gene expression was significantly (*p* < 0.05) increased in CEs from the control group challenged with LPS in comparison to the rest of the CE groups evaluated. NFkB gene expression was significantly (*p* < 0.05) diminished in CEs treated with both 25 % PRG supernatants and 50 % P-PRG supernatant in comparison to the CEs from the healthy control group and those treated with 50 % P-PRG (Fig. 4).

**Fig. 5:**
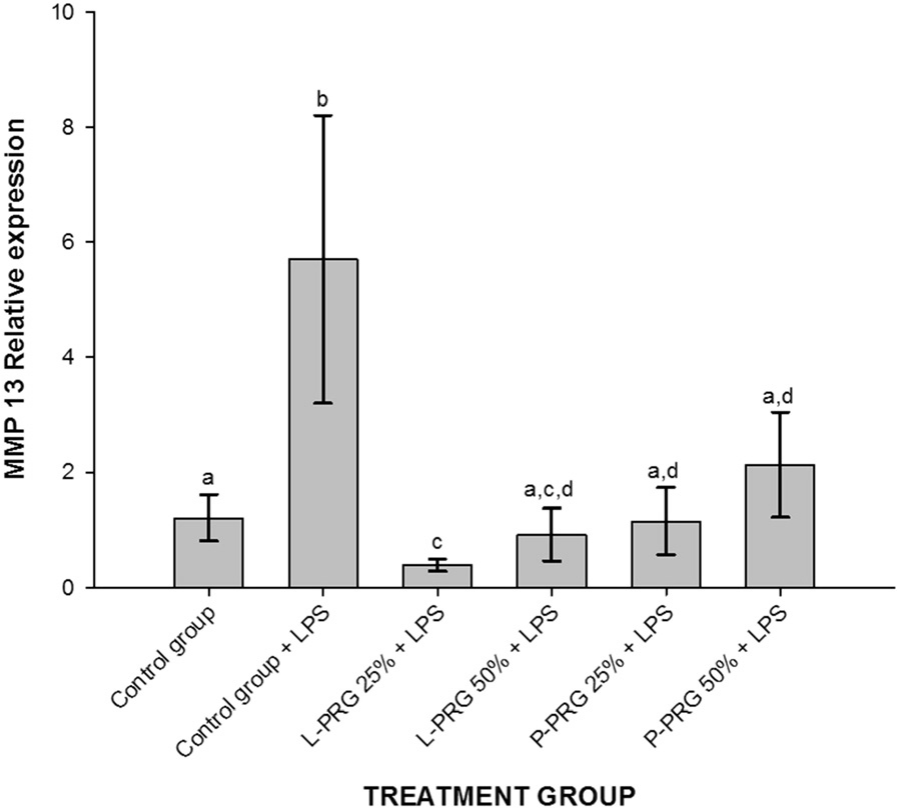
Means (± s.e.m) of MMP-13 gene expression in CE groups at 96 h ^a-b^ Different lowercase letters denote significant (*p* < 0.05) differences between the groups evaluated by the Tukey test

CEs from the healthy control group and all PRG supernatant treatments presented a significant (*p* < 0.05) decrease of MMP-13 expression (Fig. 5). However, 25 % L-PRG supernatant induced a lesser expression of this gene in CEs when compared to CEs treated with 50 % P-PRG supernatant (*p* < 0.05). No significant differences were noticed for the expression of this catabolic gene in the CEs from the healthy control group and those treated with 50 % L-PRG, and 25 and 50 % P-PRG supernatants (Fig. 5).

**Fig. 6:**
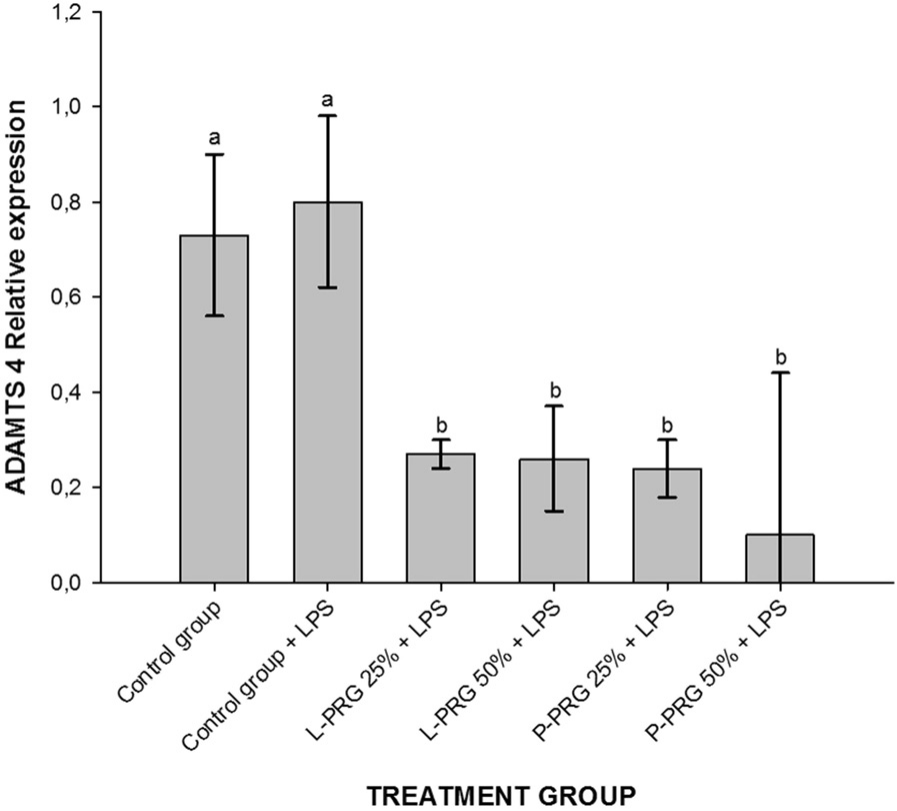
Means (± s.e.m) of ADAMTS-4 gene expression in CE groups at 96 h. ^a-b^ Different lowercase letters denote significant (*p* < 0.05) differences between the groups evaluated by the Tukey test

**Fig. 7:**
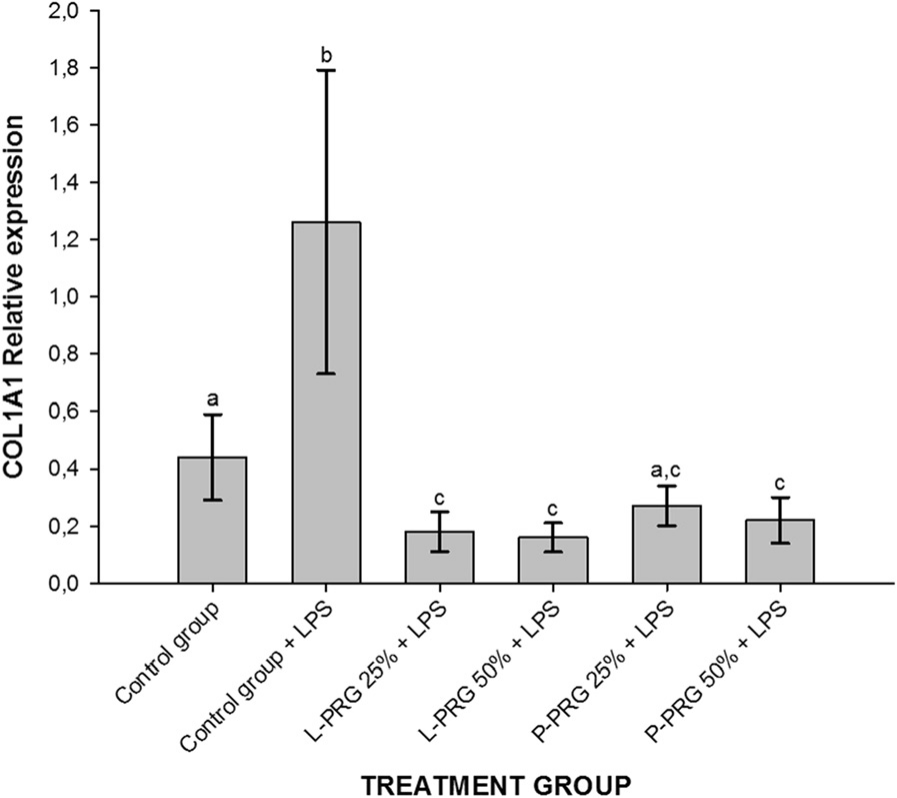
Means (± s.e.m) of COL1A1 gene expression in CE groups at 96 h. ^a-b^ Different lowercase letters denote significant (*p* < 0.05) differences between the groups evaluated by the Tukey test

**Fig. 8:**
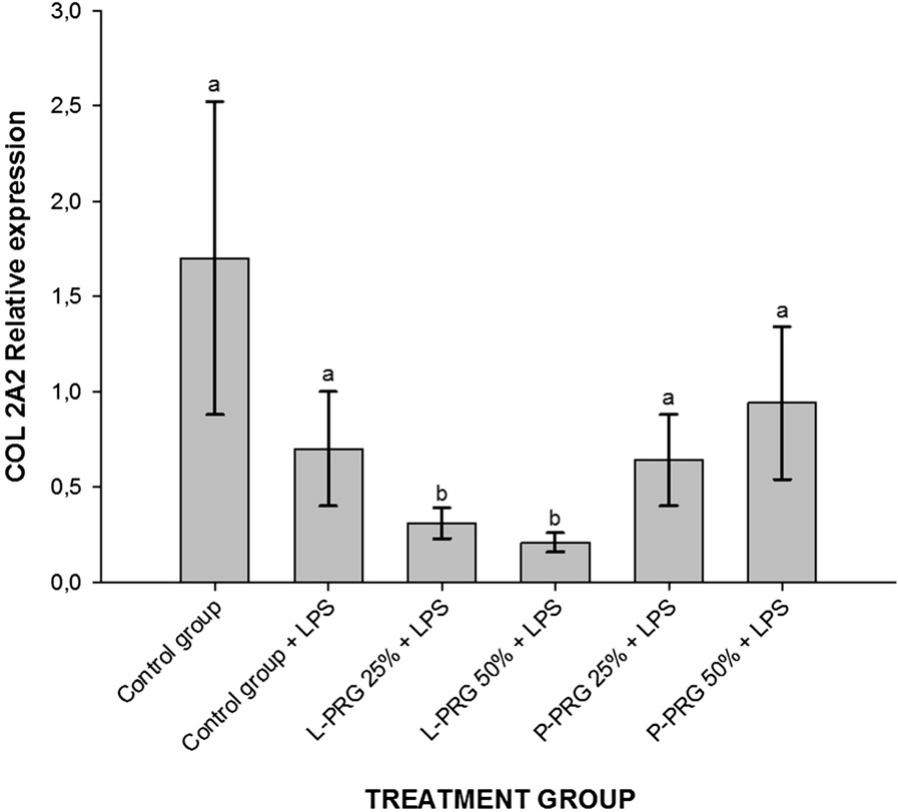
Means (± s.e.m) of COL2A1 gene expression in CE groups at 96 h. ^a-b^ Different lowercase letters denote significant (*p* < 0.05) differences between the groups evaluated by the Tukey test

All PRG supernatant treatments significantly (*p* < 0.05) decreased ADAMTS-4 gene expression in CEs when compared to CEs of both control groups (Fig. 6). Furthermore, CEs from the healthy control group and all PRG supernatant treatments showed significant (*p* < 0.05) COL1A1 gene downregulation when compared to CEs of the control group challenged with LPS (Fig. 7). L-PRG supernatants at different concentrations significantly (*p* < 0.05) diminished the COL2A1 gene expression in CEs in comparison to CEs treated with both P-PRG supernatant concentrations and the control group. Notably, there were not significant differences between these last three groups and CEs from the healthy control group for COL2A1 gene expression (Fig. 8). However, although this gene expression was statistically (*p* = 0.09) similar among CEs from both control groups, COL2A1gene expression was apparently higher in CEs from the healthy control group than in CEs challenged only with LPS (Fig. 8).

**Fig. 9:**
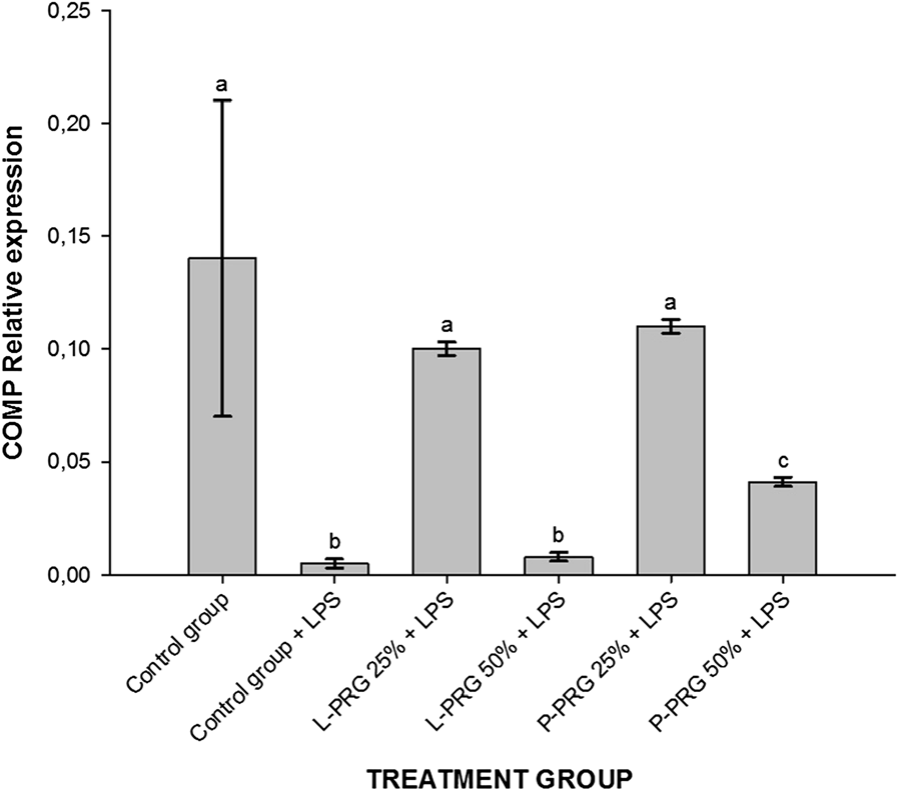
Means (± s.e.m) of COMP gene expression in CE groups at 96 h. ^a-b^ Different lowercase letters denote significant (*p* < 0.05) differences between the blood component groups obtained with the same anticoagulant by the Tukey test

COMP gene expression was significantly (*p* < 0.05) diminished in CEs from the control group challenged with LPS and those treated with the 50 % L-PRG supernatant in comparison to CEs treated with both the 25 % PRG supernatants and the 50 % P-PRG supernatant. CEs from the healthy control group and those treated with both 25 % PRG supernatants presented similar increased COMP gene expression. However, CEs treated with both 25 % PRG supernatants demonstrated a significantly higher CE gene expression when compared to CEs treated with 50 % P-PRG supernatant (Fig. 9).

## Discussion

Diverse PRP effects on cells and tissues are well recognised as depending on their varying preparations [38]. Thus, the molecular profile of a PRG supernatant is associated with the type and quantity of WBCs and PLTs initially present in PRP [25, 28]. However, after PRP is activated in a joint, it becomes a fibrin mesh or PRG that releases growth factors and cytokines and acts as an immune node inside the joint [49]. We currently believe that the cell ratio (PLT:WBC) in PRP is not as critical as the GF:GF or GF:cytokine ratios and concentrations released into the joint fluid [50]. However, we also wish to clarify that this assumption could possibly be valid for the use of PRP in joints and not for the infiltrative use of this substance in tendons, ligaments or muscles, where not only is the growth factor concentration important, but also the fibrin mesh to allow growth factor storage, cell migration and cell proliferation [51].

Strong debates continue about the ideal PLT:WBC ratio and concentration in PRP [51]. Currently, there are several studies indicating that plasma with a poor quantity of PLTs and negligible WBs could be the best “PRP” preparations to treat OA and other musculoskeletal pathologies [35, 36, 52]. However, a recent in vitro study described molecular positive effects for several commercial kits which have concentrates at different PLT:WBC ratios and are used for OA treatment [39].

Thus, we decided to conduct an independent study to evaluate the effect of two different PRP preparations (obtained by a homemade lab method) at different concentrations on cartilage histology, chondrocyte apoptotic scores, HA release and catabolic and anabolic gene expression of CEs challenged with LPS. Exposure of CEs to LPS resulted in marked changes in the expression of genes involved in matrix formation and degradation as well as inflammation of which many were reduced by the addition of both PRG supernatants at different concentrations.

The use of LPS for inducing cartilage inflammation/degeneration is a classical methodological approach to perform diverse in vitro studies by simulating a system of inflammatory/degenerative arthropaty [42, 43, 53]; although, the use of recombinant IL-1β to induce in vitro articular cartilage degradation could be more suitable [54, 55]. However, it is important to clarify that the cellular receptors for LPS and IL-1β are closely interlinked and described as the TLR-IL-1 receptor (IL-1R) superfamily of receptors. When activated they induce a series of intercellular signalling pathways that converge on the activation of the transcription NFkB resulting in pro-inflammatory cytokine expression [55, 56].

It is important to mention that the evaluation of the anti-inflammatory effect of PRG supernatants by measuring the relative expression of the NFkB gene in the present study represents a very limited approach to understanding the activity signalling of this important inflammatory process [57]. However, an in vitro study in human chondrocytes revealed that activated PRP induced the expression and production of inhibitor-ĸBα (IĸBα) (directly mediated by hepatocyte growth factor), which is responsible for the inhibition of the transcription of NFkB [58]. This finding has also been consistent for gingival fibroblasts and alveolar osteoblasts challenged with LPS and cultured with PRP preparations [59].

Notably, LPS challenge induced important structural (degenerative) changes accompanied with chondrocyte apoptosis in CEs, which were reverted or ameliorated in diverse degrees by PRG supernatants. However, the general histology score was significantly lower (better) for CEs treated with PRG supernatants, and some individual outcome parameters, such as chondrocyte necrosis, focal cell loss and SOFG uptake, were affected by some of these substances.

Chondrocyte necrosis was mostly observed in CEs treated with 25 % L-PRG and 50 % P-PRG supernatants. This finding could indicate that these substances at these specific concentrations were not able to reverse the catabolic effect mediated by endotoxin in CEs. Furthermore, it is also important to consider that both 25 % PRG supernatants presented chondrocyte apoptosis reaction similar to those observed in CEs from the control group challenged with LPS; although, CEs treated with 25 % P-PRG supernatant presented the highest score for this parameter. This last finding could possibly indicate that this substance at lower concentration is not able to reverse the cartilage catabolic effects of LPS and can induce additional chondrocyte apoptosis.

Focal cell loss was more evident for both 50 % PRG supernatants, possibly due to that chondrocytes being able to migrate inside the CEs towards the periphery through a chemotactic attractive stimulus in the culture media [60], such as the milieu of growth factors released from PRP [61–63]. In contrast, another explanation to demonstrate the cause of focal cell loss in the groups of CEs treated with higher concentrations of PRG supernatants could be attributed to chondrocyte necrosis or even chondrocyte apoptosis [64]. However, this lack of significant increase of these parameters occurred only for CEs treated with 50 % P-PRG supernatant, which could indicate that P-PRG preparations were not chondroprotective.

The SOFG uptake score was higher in CEs treated with 50 % L-PRG and 25 % P-PRG supernatants, and apparently this parameter was worst for CEs treated with 50 % L-PRG supernatant. This parameter is associated with the structural content of proteoglycans in the cartilage ECM [8], which indicate the quality and integrity of this structure. It is important to consider that 50 % L-PRG supernatant also induced downregulation of anabolic ECM genes, such as COL2A1 and COMP. Taken together, these histology and molecular findings indicate that higher concentrations of L-PRP preparations could be deleterious for osteoarthritic cartilage. One pitfall of the present research was that the release or accumulation of proteoglycans was not measured in the culture media of CEs. However, we consider that the combination of histology parameters and gene expression could be valid indicators for the degree of affection of ECM of the CEs of this study.

The combination of the general histology score, chondrocyte apoptosis findings, HA release to the culture media and the catabolic and anabolic cartilage ECM gene expression demonstrated that all PRG supernatants are “biosubstances” with mixed biological effects. In general, all PRG supernatants presented a potential anti-inflammatory effect and decreased the catabolic effects of LPS on CEs at the cellular and molecular level. However, the overall analysis of our results indicates that 25 % L-PRG supernatant (followed in order of importance by 50 % P-PRG, 25 % P-PRG and 50 % L-PRG) represented the substance with the most important anti-inflammatory and anabolic effect with lower affection of CEs at the histology level. Furthermore, 25 % P-PRG supernatant presented important anabolic effects, but it induced a more severe chondrocyte apoptosis than the other of the evaluated substances. This detrimental cell effect limits the potential clinical use of this substance at this concentration.

The present results can be complemented with the previous findings obtained by us in two in vitro studies, in which the production of anti-inflammatory and catabolic cytokines was measured in the culture media of synovial membrane explants (SME) [27] and CEs [40] challenged with LPS over 48 h and 96 h. In general, 25 % L-PRG and 50 % L-PRG supernatants presented the best anabolic and anti-inflammatory profile. However, in both studies, 25 % L-PRG supernatant produced a most significant reduction of TNF-α production and a total higher production of IL-1ra over time than 50 % L-PRG [27, 40].

Currently, we use 3-5 mL doses of PRP to treat fetlock osteoarthritis in horses with a body weight of 300-450 kg. This PRP volume has also been used for other researches with good results [22]. Notably, the complete volume capacity for an equine fetlock joint has been calculated at 12.5 ± 1.0 mL [65]. Thus, if the PRP volume used is adjusted in relation to the volume capacity of this specific joint [65], the effective concentration of PRP in clinical conditions could be near to 40 %, without considering the additional volume of synovial fluid (2-2.5 mL), which could represent a final PRP/synovial fluid concentration of 40-60 %. In line with this, the PRP dose for this joint could possibly be adjusted at 25 % using between 1.5-2.0 mL. However, this hypothesis should be probed in patients with osteoarthritis or experimentally induced synovitis.

Our findings are in line with the results of Cavallo et al. [39] and Kreuz et al.[38], who found diverse and positive effects for L- and P-PRP preparations in human osteoarthritic chondrocytes and subchondral mesenchymal progenitor cells. Conversely, our results are different from other in vitro studies [34–36], which have concluded that only P-PRP preparations induce an anabolic effect in cartilage and L-PRP preparations only induce an opposite (catabolic) joint state.

This study had several limitations. First, this is an in vitro study, which does not reflect the exact biological phenomena occurring in the osteoarthritic joint. However, this in vitro system of cartilage inflammation and degeneration could be useful to demonstrate that L- and P-PRG supernatants could act in function of their concentration and could have different mechanisms of action to induce cartilage joint anabolism and, potentially, diminish pain and inflammation. Another limitation of this study was that blood of only one animal was used to produce the PRG supernatants evaluated to avoid the great variability in the CE responses to several PRP preparations. This situation is not practical in clinical conditions because autologous PRP should be used. However, the response of each patient in particular will depend in some measure on the concentration of the cells’ proteins in PRP.

## Conclusions

This study demonstrates that both L- and P-PRP preparations at different concentrations can induce different anti-inflammatory, anti-catabolic, anabolic and even catabolic responses in an in vitro system of cartilage inflammation and degeneration.

Apparently, 25 % L-PRG supernatant presented the best therapeutic profile when compared to the other PRP preparations examined, because it presented anti-inflammatory and anabolic effects with minimal cellular and structural changes in CEs. Our results once more demonstrate that the biological variability of PRP preparations makes their application rather challenging. Additional studies in animal models and in patients with OA should be performed to know the in vivo effect of PRP preparations at different concentrations.

## Abbreviations

ADMTS-4, A disintegrin and metalloproteinase with thrombospondin motifs 4; ANOVA, analysis of variance; CEs, cartilage explants; COL1A1, collagen type I alpha 1; COL2A1, collagen type II alpha 1; COMP, cartilage oligomeric matrix protein; DMEM, Dulbecco’s modified Eagle medium; ECM, extracellular matrix; ELISA, enzyme-linked immunosorbent assay; GF, growth factor; HA, hyaluronan; IL-1, interleukin 1; L-PRG, leukoconcentrated platelet-rich gel; L-PRP, leukoconcentrated platelet-rich plasma; LPS, lipopolysaccharide; MMP-13, matrix metalloproteinase 13; NFkβ, nuclear factor kappa beta; OA, osteoarthritis; PDGF-BB, platelet derived growth factor isoform BB; PLT, platelet; P-PRG, pure platelet-rich gel; P-PRP, pure platelet-rich plasma; PRG, platelet-rich gel; PRP, platelet-rich plasma; RBC, red blood cell; TGF-β_1,_ transforming growth factor beta-1; TNF-α, tumor necrosis factor alpha; WBC, leukocyte.
